# Acquired hemophilia A (AHA): underreported, underdiagnosed, undertreated medical condition

**DOI:** 10.1186/s43162-021-00074-9

**Published:** 2022-01-25

**Authors:** Doaa M. El Demerdash, Alia Ayad, Noha Tawfik

**Affiliations:** Internal Medicine Department, Kasr Al-Ainy Hospital, Faculty of Medicine, Cairo University, Cairo, Egypt

**Keywords:** Hemophilia, Acquired, Bleeding, Prolonged APTT

## Abstract

**Background:**

Acquired hemophilia A (AHA) is a rare acquired bleeding disorder occurred due to the formation of inhibitory antibodies neutralizing endogenous factor VIII.

**Main body:**

About half the cases are idiopathic. Symptoms include severe and unexpected bleeding that could be life-threatening. High index of suspicion should be raised when unexplained subcutaneous or post-surgical bleeding with isolated prolonged APTT.

**Conclusions:**

Acquired hemophilia A is a rare underdiagnosed underreported acquired hemostatic disorder that presents with sudden usually life-threatening bleeding; it is crucial to raise awareness and suspicion index of clinicians for early diagnosis and treatment to avoid morbidity and mortality.

## Background

Acquired hemophilia A (AHA) is a rare bleeding disorder; it is an underreported condition because of the severity of AHA at presentation with life-threatening bleeding especially in the elderly in whom other bleeding conditions may coexist; also, it is underdiagnosed due to rarity and the unawareness of the condition which sometimes presented to surgical wards, and this may lead to delayed diagnosis hence worse outcome [1]. Our aim in this review article is to raise the awareness of the underreported acquired cause of bleeding.

## Main text

### Acquired hemophilia recurrent scenario

To clarify this recurrent scenario here, one of our cases, a female patient, 32 years old, G2P1, presented to the obstetric department for normal vaginal delivery without any previous known medical problems. She had hemoglobin (Hb%) of 11 g/dl with a normal baseline coagulation profile. Her puerperium was complicated by excessive vaginal bleeding with a drop in her hemoglobin from 11 to 6.8 g/dl. Hysterectomy was performed for the persistent vaginal bleeding that was complicated by intra-abdominal hemorrhage. Her condition continued to deteriorate; the gynecologist feels that something was wrong with no obvious obstetric cause of bleeding, and she was referred to ICU with a diagnosis of DIC. Her labs on admission are summarized in Table 1; the striking result noticed was an isolated prolonged APTT. The striking result noticed was an isolated prolonged aPTT, with normal platelets count, normal PT, and slightly elevated d-dimer and FDP, and that is why the diagnosis of DIC was not convincing

**Table 1:** Initial laboratory workup of an acquired hemophilia case

Laboratory tests	Patient’s value	Normal value
PC	80%	70–100%
Platelet count	262,000 mm^3^	130–400 mm^3^
aPTT	68.4 s	25.1–34.7 s
FDP	20 mg/L	<10 mg/L
D-Dimer	1 μg/mL	<0.5 μg/mL

Acquired hemophilia A (AHA) which is a severe acquired rare bleeding disorder. It is a rare bleeding disorder; in the reported cases, only 1–1.5 per million persons are affected yearly [2], AHA characterized by suddenly appearing autoantibodies (inhibitors) that partially or completely neutralize the activation or function or accelerate the clearance of factor VIII [3].

Although it is like well-known congenital hemophilia in lacking factor VIII activity and hence bleeding tendency, but it is totally different from the well-known congenital hemophilia as It affects both males and females without previous family or personal history of bleeding while congenital hemophilia affects only males with a long history or family history of bleeding [4].

AHA incidence is typically observed in old age (> 60 years old) but another peak is noticed in middle age females especially with reported postpartum AHA cases [5].

Etiology is unknown in half of the reported cases but reported associated conditions (Table 2) in the other half were solid and hematological malignancies, autoimmune conditions such as SLE, dermatological conditions as pymphygus, drug-induced, pregnancy, and recently reported with COVID-19 and post COVID vaccines [11, 12].

**Table 2:** Conditions may be associated with acquired hemophilia A

Conditions may be associated with acquired hemophilia A	
**Autoimmune disorders** **[**6**]**	Rheumatoid arthritis Systemic lupus erythematosus Multiple sclerosis Temporal arteritis Sjögren syndrome Autoimmune hemolytic anemia Goodpasture syndrome Myasthenia gravis Graves disease Autoimmune hypothyroidism
**Malignancies** **[**7**]**	**Solid tumors** (prostate, lung, colon, pancreas, stomach, bile duct, head and neck, cervix, breast, melanoma, kidney) **Hematologic malignancies** Chronic lymphocytic leukemia Non-Hodgkin lymphoma Multiple myeloma Waldenström macroglobulinemia Myelodysplastic syndrome Myelofibrosis AML (M6)
**Dermatologic disorders** **[**8**]**	Bullous Pemphigoid Psoriasis vulgaris Vitiligo Squamous cell carcinoma
**Allergic drug reactions** **[**9**]**	Penicillin and its derivatives Sulfamides Phenytoin Chloramphenicol Methyldopa Depot thioxanthene Interferon alfa Fludarabine Bacille Calmette-Guérin (BCG) vaccination Desvenlafaxine
**Postpartum AHA** **[**10**]**	
**Other conditions**	Inflammatory bowel disease, ulcerative colitis Respiratory diseases (e.g., asthma, chronic obstructive pulmonary disease) Diabetes Acute hepatitis B infection Acute hepatitis C infection

The cause of bleeding tendency is the same in both classic and AHA which decrease in the factor VIII activity. However, the clinical manifestation is not identical; in classic congenital hemophilia, usually, spontaneous bleeding into joints is typical, while in AHA, type of bleeding is different which is massive subcutaneous blood extravasations and mucosal hemorrhages [13].

The bleeding phenotype in AHA is variable ranging from mild to life-threatening bleeding, and there is poor correlation between FVIII level and inhibitor titer at presentation with bleeding severity unlike the congenital hemophilia bleeding phenotype [14].

The mortality rate is 41% if AHA patients are not treated: within the 1st week of presentation, mortality occurs due to GIT and lung bleeding Later, mortalities are usually from intracranial and retroperitoneal hemorrhages [15].

It is worth to be noted that 10% of patients do not present with bleeding and accidentally discovered with laboratory workup; therefore, a prolonged APTT should never be ignored prior to invasive procedures [16].

### Post-partum acquired hemophilia A

We must focus on the entity which is postpartum acquired hemophilia in which there is spontaneous development of autoantibodies (inhibitors) against factor VIII during the peripartum period; it is reported in 7–21% of AHA cases [10]. Documented cases of postpartum AHA in EACH2 registry which included 501 AHA cases from 117 hemophilia centers in 13 European countries was 42 (8.4%). Mortality due to hemorrhage in these cases varies between 12 & 22% [17]. It Should be suspected in postpartum or pregnant patients who hemorrhage for no apparent cause and who have no previous history of bleeding disorders. Post-partum AHA usually appear after the 1st pregnancy in 80% of the cases. Most reported cases arise from 1 to 4 months after delivery, but some cases presented up to 1 year after delivery [18].

It commonly presents as severe ecchymosis, soft tissue hematomas, and severe life-threatening hemorrhage. Vaginal bleeding could be the presenting symptom if the inhibitor appears early in the course, so it is usually misdiagnosed by an obstetrician especially with no previous bleeding or family history in presented females with postpartum unexplained bleeding. Intraplacental transfer of antibodies and intracerebral hemorrhage in neonates are reported in few cases [19].

### AHA laboratory workup

Laboratory workup of a suspected case of AHA especially in the setting of unexplained bleeding associate with an isolated prolonged aPTT without previous medical or family history of bleeding should include measuring factor VIII activity and its inhibitor in those patients.

### Initial workup

Individuals present with new-onset bleeding without previous history of bleeding disorders. Labs will show an isolated increase in aPTT with a normal PT, platelet count, and thrombin time. This points to either a deficient factor VIII or the presence of a factor VIII Inhibitor.

Therefore, a mixing study must be ordered to determine the cause. If there is a factor deficiency, the results will show a correction of PTT. If there is a factor inhibitor present, the PTT will remain elevated and uncorrected or partially corrected [2].

### Confirmation test

**Fig. 1:**
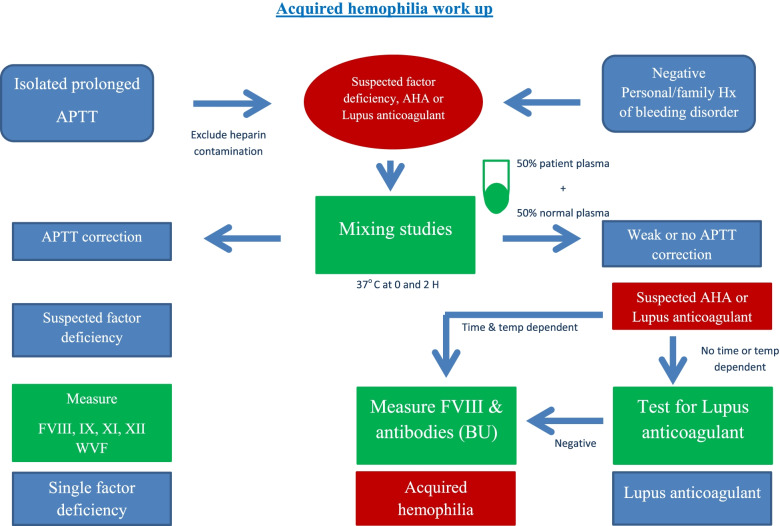
Workup of a case of AHA

Factor VIII activity test to exclude the presence of antiphospholipid antibodies and Bethesda assay to measure the strength of the inhibitors in the plasma, expressed in Bethesda units [20]. Workup of a case with AHA is summarized in Fig. 1.

### Why acquired hemophilia A is undermanaged?

AHA is a rare but potentially lethal disease this make it underreported, no comparative clinical studies are available in AHA, the severity of the clinical condition of patients at presentation, and the decisions are often based on the clinical experience of treating physicians, all these factors lead to undermanagement of AHA [21].

Management of AHA is representing a medical challenge from its diagnosis to its treatment, the early initiation of Therapy results in higher efficacy. The only parameter that differed between patients who responded to treatment and those who did not was a delay in time to treatment [22].

Optimal management included the use of “bypass” agents when active bleeding presents, altogether with active inhibitor eradication through immunosuppressant drugs, the 2 lines of treatment should be started aside with most importantly treatment of any underlying condition, e.g., autoimmune disease. Further recommendations should include avoidance of invasive procedures that may induce bleeds even sampling of blood or inserting IV cannula [23].

### Control bleeding if present

Lines to control the acute bleeding includes:

- Bypassing agents: Recombinant Factor VIla (rFVIIa) or FEIBA, activated prothrombin complex concentrate (APCC)
- Recombinant porcine factor VIII rpFVIII (susoctocog alfa)

Registries data did not show a clear efficacy or safety benefit of one drug over the others.

- Recombinant Factor VIla (rFVIIa)

It is given as 90 μg/kg bolus at every 2 h, Cases of mild bleeding, one or two doses of rFVIIa may be sufficient. Once hemostasis has been achieved, the dose interval can be increased successively to every 4, 6, 8, or 12 h when indicated. rFVIIa controls bleeding at the site of vascular injury only. Its safety profile includes that thrombotic events of rFVIIa <1% with an ~800,000 doses, it is not contraindicated for use with anti-fibrinolytics, it is a pure FVII, with no risk of anamnesis, and also it is a recombinant product with no risk of viral transmission [24].

- FEIBA, an activated prothrombin complex concentrate (aPCC)

It includes factor II, VII, IX, X, and small amount of factor VIIIa. Also, it includes natural anticoagulant (protein C and S) in physiological balance. aPCC doses in the range of 50–100/kg units every 8–12 h are given by intravenous infusion. it is important not to exceed a total of 200 units/kg within a 24-h period as this may be associated with a risk of VTE or DIC. We must point out that tranexamic acid should not be given together with this agent [25, 26].

- Recombinant porcine factor VIII rpFVIII (susoctocog alfa)

It could be given in cases of AHA as human anti-FVIII autoantibodies have low cross-reactivity with porcine FVIII, it could be given in Patients with AHA with a serious bleed but it should be excluded if they had an anti-rpFVIII inhibitor titer >20 BU.

rpFVIII dose of 200 U/kg, followed by further doses to maintain trough levels of factor VIII >50% .close monitoring of FVIII activity during therapy is recommended to avoid the theoretical risk of thrombosis while there were no thromboembolic events reported [27].

### Eradications of inhibitors by immunosuppressants

In all patients with AHA, they should receive immunosuppressants to eradicate inhibitors in form of corticosteroids alone or plus either rituximab or cytotoxic agents, while speaking about steroid therapy mentions that oral prednisone is the preferred one [28]. If FVIII ≥1 IU/dL and inhibitor titer ≤20 BU at baseline receive 1st line treatment with corticosteroids alone for 3–4 weeks is recommended, but If FVIII <1 IU/dL or inhibitor titer >20 BU it is suggested combining corticosteroids with rituximab or a cytotoxic agent for 1st line therapy. If no response to 1^st^ line therapy 2nd line therapy with rituximab or a cytotoxic agent, whichever was not used during first-line therapy is suggested, immune tolerance induction in AHA. Or high-dose intravenous immunoglobulins for inhibitor eradication in patients with AHA are not recommended [21].

Because relapse has been reported in approximately 1 in 5 patients after immunosuppressive therapy is discontinued. The assessment of the response requires follow-up of factor VIII activity and inhibitors assay. It is recommended of using FVIII:C monitoring monthly during the first 6 months, every 2–3 months up to 12 months, and every 6 months during the second year and beyond, if possible [21] (Table 3).

**Table 3:** Immunosuppressive therapy for AHA

**FVIII** ***≥*** **1% and < 20 BU/ml**	Steroids alone 3–4 weeks
	If no response add cyclophosphamide or rituximab
**FVIII** ***<*** **1% or > 20 BU/ml**	Steroids and cyclophosphamide or rituximab 3–4 weeks
	If no response add cyclophosphamide or rituximab

Table 2 summarizes 2020 International recommendations of immunosuppressive therapy in the treatment of acquired hemophilia A.

### Back to presented scenario

workup of the patient was continued as a case presented with isolated prolonged aPTT. Laboratory results confirm the diagnosis of postpartum AHA as demonstrated below.

Diagnosis of postpartum AHA was confirmed by doing mixing studies for aPTT which was not corrected by adding normal plasma which confirm the presence with of inhibitor, absence of antibodies for antiphospholipid altogether with a reduced level of factor VIII, and presence of FVIII antibodies in our case confirm the final diagnosis of postpartum AHA (Table 4).

**Table 4:** Continued laboratory workup of an acquired hemophilia case

aPTT Mixing studies	67 s	25.1–34.7 s
LA	Negative	Negative
Factor VIII rate	4%	70–150%
Factor VIII inhibitor level	10 BU	<0.6 BU
Factor IX rate	103.9%	70–120%
von Willebrand factor	89.5%	50–160%

Her bleeding was life-threatening and massive due to late diagnosis and management, also it was aggravated by the surgical intervention (hysterectomy), as we must point out that any surgical intervention may aggravate antibodies formation and hence the severe bleeding [28].

To stop bleeding, rFVIIa was given 90 mic/kg every 2 h then gradually spaced to be every 6 h, 12 h, and 24 h (12 doses were needed till regression), prednisone 1mg/kg/day was added (4 weeks duration) to eradicate inhibitor, her course was stable apart from intermittent symptoms and prolongation of APTT which mean persistent inhibitor rituximab was added in a dose of 375 mg/m^2^ for 4 doses, and resolution of inhibitors was achieved after 3 weeks of initiation of added rituximab.

## Conclusions

Acquired hemophilia A is a rare underdiagnosed underreported acquired hemostatic disorder that presents with sudden usually life-threatening bleeding, it is crucial to raise awareness and suspicion index of clinicians for early diagnosis and treatment to avoid morbidity and mortality.

## Data Availability

Not applicable

## Abbreviations

AHA: Acquired hemophilia A
APCC: Activated prothrombin complex concentrate
APTT: Activated partial thromboplastin time
BCG: Bacille Calmette-Guerin
BU: Bethesda unit
COVID: Coronavirus sisease
DIC: Disseminated intravascular coagulation
EACH2: European Acquired Hemophilia Registry
FDP: Fibrin degradation products
FEIBA: Factor eight inhibitor bypass activity
GIT: Gastrointestinal tract
ICU: Intensive care unit
rPFVIII: Recombinant porcine factor VIII
SLE: Systemic lupus erythematosus
VTE: Venous thromboembolism
